# Secondary bile acids mediate high-fat diet–induced upregulation of R-spondin 3 and intestinal epithelial proliferation

**DOI:** 10.1172/jci.insight.181492

**Published:** 2024-06-10

**Authors:** Ji-Yao Li, Merritt Gillilland, Allen A. Lee, Xiaoyin Wu, Shi-Yi Zhou, Chung Owyang

Original citation *JCI Insight*. 2022;7(19):e148309. https://doi.org/10.1172/jci.insight.148309

Citation for this retraction: *JCI Insight*. 2024;9(11):e181492. https://doi.org/10.1172/jci.insight.181492

The University of Michigan recently notified *JCI Insight* that a research misconduct investigation committee found that there was falsification and/or fabrication of RT-PCR data in Figure 1C. In accordance with the institutional recommendation, *JCI Insight* is retracting this article.

## Version 1 04/22/2024

Electronic publication

## Version 2 06/10/2024

Electronic publication

